# Supplementary material for: Inferring Parameters of Pyramidal Neuron Excitability in Mouse Models of Alzheimer’s Disease Using Biophysical Modeling and Deep Learning
Source: Bull Math Biol. 2024 Mar 25;86(5):46. doi: 10.1007/s11538-024-01273-5 (PMC10963524; doi:10.1007/s11538-024-01273-5)
Supplement: Supplementary file 1 — (pdf 12855 KB) [file 11538_2024_1273_MOESM1_ESM.pdf]

Supplementary Information

Saghafi et al. (2024). *Inferring parameters of pyramidal neuron excitability in mouse models of Alzheimer's disease using biophysical modeling and deep learning*

1 Mechanistic Model Parameters

Table 1 Parameters of CA1 pyramidal neuron model. For most parameters, we used the values given in Nowacki et al. [1] paper. Parameter values for the hyperpolarization-activated potassium current (I_H) are taken from Booth et al. [2]. Optimized parameter values for the maximal conductances and transient sodium half-activation that we obtained through differential evolution (DE-MG-Vmnat) are shown in parentheses, and were used as the default μ_p values when generating the training and target datasets.

Parameter	Value	Units	Parameter	Value	Units
C_m	1	μF/cm^2	V_{m_H}	-102	mV
E_{Na}	60	mV	V_{n_H}	-102	mV
E_{Ca}	90	mV	$k_{m_{Na_T}}$	5	mV
E_K	-85	mV	$k_{h_{Na_T}}$	-7	mV
E_H	-30	mV	$k_{m_{Na_P}}$	3	mV
E_L	-65	mV	$k_{m_{Ca_T}}$	5	mV
g_{Na_T}	65.0 (7.2603)	μS/cm^2	$k_{h_{Ca_T}}$	-8.5	mV
g_{Na_P}	0.1 (0.0423)	μS/cm^2	$k_{m_{Ca_H}}$	5	mV
g_{Ca_T}	0.6 (0.067)	μS/cm^2	$k_{h_{Ca_H}}$	-7	mV
g_{Ca_H}	0.74 (1.5208)	μS/cm^2	$k_{m_{K_{DR}}}$	11.4	mV
$g_{K_{DR}}$	9.5 (12.505)	μS/cm^2	$k_{h_{K_{DR}}}$	-9.7	mV
g_{K_M}	0.8 (3.3837)	μS/cm^2	$k_{m_{K_M}}$	10	mV
g_H	0.05 (0.0503)	μS/cm^2	k_{m_H}	-13	mV
g_L	0.02 (0.0035)	μS/cm^2	k_{n_H}	-6	mV
$V_{m_{Na_T}}$	-37 (-60.00)	mV	$\tau_{m_{Ca_T}}$	2	ms
$V_{h_{Na_T}}$	-75	mV	$\tau_{h_{Ca_T}}$	32	ms
$V_{m_{Na_P}}$	-47	mV	$\tau_{m_{Ca_H}}$	0.08	ms
$V_{m_{Ca_T}}$	-54	mV	$\tau_{h_{Ca_H}}$	300	ms
$V_{h_{Ca_T}}$	-65	mV	$\tau_{m_{K_{DR}}}$	1	ms
$V_{m_{Ca_H}}$	-15	mV	$\tau_{h_{K_{DR}}}$	1400	ms
$V_{h_{Ca_H}}$	-60	mV	$\tau_{m_{K_M}}$	75	ms
$V_{m_{K_{DR}}}$	-5.8	mV	τ_{m_H}	15	ms
$V_{h_{K_{DR}}}$	-68	mV	τ_{n_H}	210	ms
$V_{m_{KM}}$	-30	mV	p	0.85	-

2 Supplementary Methods

2.1 Differential Evolution (DE) - global optimization

Differential evolution is a type of stochastic global optimization and a population-based search technique first introduced by Storn and Price, 1997 [3, 4]. This method is comprised of four different steps, including *Initialization*, *Mutation*, *Crossover*, and *Selection.*

Initialization - a population of individuals, which is also known as the first generation or parents, is often defined by drawing a population number randomly from a uniform distribution to explore the objective landscape. In this context an individual is essentially an ordered set of parameters.

Mutation - a new vector (a.k.a donor vector) is introduced in this step by a linear combination of three random vectors from the current generation but not the one which we are going to compare and substitute with in the next generation (i.e. target vector), this means at least four members are needed for applying this method.

Crossover - the recombination of donor vector with the best member of the previous generation with a probability rate bring both exploration and exploitation to the search and lead the system get out of the local minima. *Selection* - during the selection step, we have a new vector, which is a candidate for going to the new generation in replace of the target vector, if it satisfies certain conditions, and it has a better score [1] than the target vector. In this way, after each generation we might have better members or in the worst-case scenario all members could be the same as the previous generation without any change but none of them getting worse than before.

The Differential Evolution Algorithm that we used in this project known as **classic DE** in [3]. It is also referred to as DE/rand/1/bin which means the base vector is *randomly* selected and only *1* vector difference is added in the mutation part. Finally, the donated parameters in the crossover section follow a *binomial* distribution.

[1]The score could be the output of the objective function and depends on the minimization or maximization problem. The new candidate will be either rejected or accepted for the next generation.

2.2 Sobol indices - global sensitivity analysis

Sensitivity Analysis (SA) [5–9] addresses the question of how much the output of a model changes given a change in the input. There are two main classes of sensitivity analysis, *local* and *global*. In local SA, the goal is to deal with sensitivity in the neighborhood of a particular parameter value. Taking a derivative is an example of local sensitivity analysis as it only consider the behavior of the system near a single point. Global SA, on the other hand, focuses on the variability of model outputs throughout parameter space. Global SA provides more information about the system and because of that it is often preferred, but if the system is too large the computational cost of this technique can be prohibitive. There are several different global SA approaches, including linear methods, tree-based methods, regionalized sensitivity analysis (also known as Monte Carlo filtering methods), and variance-based techniques [10, 11]. Sobol SA is a variance-based technique that considers the contribution of the input parameters to the variance of the system outputs. In Sobol SA we typically use two measures, the first order index and total effect index. The first order index is the contribution of an individual parameter to the response variance without considering any interactions with the other parameters. The total effect index is the contribution of an individual parameter to the response variance that does consider interactions with the other parameters.

Suppose we have a function of f that maps the vector of variables $X = (X_1, X_2, \cdots, X_p)$ to some quantity of interest. We assume X has some known probability distribution, this correspond to certain parameters in the model which this probability distribution reflect the level of uncertainty in them. The goal is to determine the sensitivity of f to X, under the assumption that f is square integrable.

$$\begin{aligned} f \colon \mathbb{R}^p &\to \mathbb{R} \\ X &\mapsto f(X) \end{aligned}$$

If f be square integrable (i.e. $f(x) \in L^2$), we can decompose the above function as the sum of the functions of only $1, 2, \cdots, p$ parameters:

$$f(X) = f_0 + \sum_{i=1}^{p} f_i(X_i) + \sum_{1 \leq i < j \leq p} f_{i,j}(X_i, X_j) + \cdots + f_{1,2,\cdots,p}(X_1, X_2, \cdots, X_p) \tag{1}$$

where:

$$\begin{aligned} f_0 &= \mathbb{E}[f(X)] \\ f_i(X_i) &= \mathbb{E}[f(X) \| X_i] - f_0 \\ f_{i,j}(X_i, X_j) &= \mathbb{E}[f(X) \| X_i, X_j] - f_i(X_i) - f_j(X_j) - f_0 \end{aligned}$$

If $X_1, X_2, \cdots, X_p$ are statistically independent then all f satisfying the orthogonality property, which includes:

$$\begin{aligned} Var(f(X)) &= \sum_{k=1}^{p} D_k(X_k) + \sum_{1 \leq k \leq k' \leq p} D_{k,k'}(X_k, X_{k'}) + \cdots + D_{1,2,\cdots,p} \\ &= \sum_{u} D_u(X_u), \quad u \subset \{1, 2, \cdots, p\} \end{aligned}$$

where:

$$D_k(X_k) = Var\left[f_k(X_k)\right], \quad D_{k,k'}(X_k, X_{k'}) = Var\left[f_{k,k'}(X_k, X_{k'})\right]$$

$$D_0 = Var\left[f_0\right] = 0, \quad \text{and} \quad u = \{i_1, i_2, \cdots, i_s\}, \quad 1 \leq s \leq p$$

This means the total variance of the response $f(X)$ can be written as the sum of partial variances. By defining the total variance of the response $f(X)$ that can be attributed to input parameter X_k as the ratio of $Var\left[\mathbb{E}[f(X) \| X_k]\right] / Var(f(X))$, the Sobol index for a subset **u** (i.e. $u \subset \{1, \cdots, p\}$) can be defined as the ratio between the contribution given by the interaction among the components of **u** for the model variance, and the total variance itself. Thus, the Sobol index for a subset **u** can be written as:

$$S_u = \frac{D_u(X_u)}{Var(f(X))}, \quad \sum_{u \subset \{1,\cdots,p\}} S_u = \frac{\sum_u D_u(X_u)}{Var(f(X))} = \frac{Var(f(X))}{Var(f(X))} = 1 \tag{2}$$

As was mentioned before, in Sobol SA, two measures (i.e. the first order index and the total index) are usually computed. The first order index refers to the contribution of any one parameter to the output variance and can be defined as:

$$S_i = \frac{D_i(X_i)}{Var(f(X))}, \quad i = 1, \cdots, p.$$

The total index refers to the contribution of all subsets with more than one parameter to the output variance, which means:

$$\begin{aligned} S_T &= \sum_{1 \le i \le j \le p} S_{i,j} + \cdots + S_{1,\cdots,p} = \sum_{u \subset \{1,\cdots,p\}} S_u \\ &= 1 - S_i, \quad i \in u. \end{aligned}$$

The first order index (i.e. S_i) describes the impact of X_i individually on the defined output, whereas, the total index (i.e. S_T) represents the effect of X_i along with the interaction of other input variables. A high value for either of these two indices suggests that X_i, either alone or in conjunction with other input variables, has a considerable overall impact on the output space.

2.3 Markov Chain Monte Carlo - MCMC

The ***Monte Carlo*** method [12, 13] is a stochastic technique that aims to calculate numerical results from many random samples. In other words, if a method uses random numbers to solve a problem, that method is a type of Monte Carlo method. By employing this randomness, it is possible that one can address some problems that, in theory, may have deterministic solutions that are hard to obtain. In order to draw some samples from a known distribution, this method repeatedly produces some random samples coming from that distribution. By repeating this process, eventually in the long run all the samples come from the desired distribution. One of the main issues with using a Monte Carlo method is that is a

memoryless process, which can lead to a very long and time consuming process to draw samples from a complex distribution. In other words, if one sample comes from a high density region of the distribution, there is no guarantee that the next candidate will also come from the same highly probable region. A ***Markov Chain*** is a sequence of events where the probabilities of the future depend only on the present [14, 15]. By incorporating a Markov process into the Monte Carlo method, which is known as ***Markov Chain Monte Carlo (MCMC)***, information about the previous step affects the next proposal candidate, and the process of sampling from complex distributions is sped up. The Metropolis Hastings algorithm (Algorithm 1) is a particular MCMC method that performs a random walk through the probability distribution to try to evaluate the values with higher probability. The main idea behind Metropolis-Hastings is that by running this algorithm for a good amount of iterations, the random walk procedure helps the Monte Carlo method to draw samples from the stationary distribution, which is the posterior distribution or the target distribution. The important fact here is that based on this algorithm, the acceptance probability that comes from the density of the model output is computed using a Gaussian mixture model that is fit to the target dataset.

Algorithm 1 Markov Chain Monte Carlo method - Metropolis Hastings

Define $f(x)$ as the target distribution, N as the total number of iterations, x_i is the current value and $q(x\|x_i)$ be a proposal distribution. This proposal distribution can be symmetric or asymmetric in this method. Define x_0 randomly from its defined range (i.e. $x_0 = x_l + rand(0,1) \times (x_u - x_l)$).

while Iter $<$ N **do**
  $x^\star \sim q(x\|x_i)$, proposed candidate
  $\rho = min\left\{1, \frac{f(x^\star)q(x_i\|x^\star)}{f(x_i)q(x^\star\|x_i)}\right\}$, $u \sim U(0,1)$,
  if $u < \rho$
    $x_{i+1} = x^\star$
  else
    $x_{i+1} = x_i$
  end
  Iter = Iter + 1
end

2.4 Jensen Shannon Divergence

$$\mathrm{JSD}(p\|q) = \frac{1}{2}\left\{\int p(x)\log\left(\frac{p(x)}{M(x)}\right)dx + \int q(x)\log\left(\frac{q(x)}{M(x)}\right)dx\right\},$$

where

$$M = \frac{p+q}{2}.$$

2.5 Cohen's *d* Effect Size

The difference between two means divided by the data's standard deviation is known as Cohen's d effect size and it can be computed based on the following formula where $s_{1,2}$ are the standard deviation and $n_{1,2}$ are the sample sizes of two different dataset:

$$d = (\bar{x_1} - \bar{x_2})/\sqrt{\frac{(n_1-1)s_1^2 + (n_2-1)s_2^2}{n_1+n_2-2}}$$

where

$$s_1 = \frac{1}{n_1-1}\sum_{i=1}^{n_1}(x_{1,i} - \bar{x_1})^2, \quad s_2 = \frac{1}{n_2-1}\sum_{i=1}^{n_2}(x_{2,i} - \bar{x_2})^2$$

Table 2 **Effect size classifications [16, 17].**

Effect size	Very small	Small	Medium	Large	Very large	Huge
d	0.01	0.20	0.50	0.80	1.20	2.0

2.6 Generative Adversarial Networks

The basic GAN consists of two artificial neural networks, a Generator G and a Discriminator D, that compete with each other in a minimax game (Fig. S1). G tries to produce fake samples that are as close as possible to real samples that come from some distribution, and D tries to distinguish real samples from fake samples. Training the GAN is an iterative process through which G gets better at fooling D, and D gets better at identifying fake samples. The first step in training the GAN is to create a training dataset (referred to as real samples) by simulating the mechanistic model with parameter sets x drawn from a uniform distribution

$P_{data}(x)$. Then we initialize G with a base distribution $P_z(z)$ which is a random variable with typically a Gaussian distribution. These parameter sets $G(z) \sim P_G$, referred to as fake samples, are passed to D along with the real samples. For a given sample x or $G(z)$, D outputs a probability $\hat{y} = D(x)$ or $\hat{y} = D(G(z))$, referred to as a reconstructed label, indicating whether it thinks the sample is real ($\hat{y} > 0.5$) or fake ($\hat{y} < 0.5$). If D is correct (i.e. $\hat{y} = D(x) > 0.5$ or $\hat{y} = D(G(z)) < 0.5$), then the weights and biases (ω, β) of the D network will remain fixed, but (ω, β) of the G network will be adjusted through backpropagation (referred to as fine-tuning in Fig. S1). If D is incorrect (i.e. $\hat{y} = D(x) < 0.5$ or $\hat{y} = D(G(z)) > 0.5$), then (ω, β) of D are adjusted while (ω, β) of G remain fixed. In practice, convergence of G and D one at a time would not only be time consuming, but also lead to instability due to a vanishing gradient for the loss function of the Generator. Therefore, in this case the weights are adjusted after computing the loss function from the outputs of D and G over each mini-batch. As a result, both G and D are being trained simultaneously, and they are converging gradually.

Derivation of the objective function for the GAN

Cross-entropy is a measure from the field of information theory which calculates the difference between two probability distributions and is defined by the following equation:

$$L(\hat{y}, y) = [y \log(\hat{y}) + (1 - y) \log(1 - \hat{y})] \tag{3}$$

where $\hat{y}$ is the reconstructed label $D(x)$ or $D(G(z))$ and y is the actual label for the sample. Therefore, the corresponding label for a real sample (i.e. a sample coming from $P_{data}(x)$) is $y = 1$ and the reconstructed label (i.e. the output of the Discriminator) is $\hat{y} = D(x)$. By substituting these labels for the real samples into the equation (3) we can get:

$$L(D(x), 1) = \log(D(x)) \tag{4}$$

Likewise the data coming from the Generator has the real label $y = 0$ and the reconstructed label is $\hat{y} = D(G(z))$, therefore, by substituting these expressions for the fake samples into Eqn. (3)

we end up with:

$$L(D(G(z)), 0) = \log(1 - D(G(z))). \tag{5}$$

Figures S2A and S2B are the visualization of Eqns. (4) and (5), respectively. Since the output of the Discriminator is a probability, in both of these panels we only consider the region between zero and one on the $x-$axis (which represents $D(x)$ or $D(G(z))$). In panel A, $\log(D(x))$ is an increasing function of $D(x)$. If we have a strong Discriminator, then we expect $D(x) \sim 1$ for a real sample. In panel B, the $x-$axis represents $D(G(z))$, and the expectation for a strong Discriminator would be $D(G(z)) \sim 0$ for a real sample. As these two points are close to the maximum of both Eqns. (4) and (5), the objective function for the Discriminator is:

$$\underset{D}{max} \left\{ \log D(x) + \log(1 - D(G(z))) \right\}. \tag{6}$$

The same logic also holds in the case of having a strong Generator, the only difference here is that we are going to minimize Eqn. (5), which corresponds to Fig. S2B. Having a very strong Generator means it is able to fool the Discriminator easily. This means the Discriminator will erroneously return a high probability even for a fake sample, i.e. $D(G(z)) \sim 1$. This point is the minimum value of Eqn. (5). Thus, the objective function for the Generator is:

$$\underset{G}{min} \left\{ \log(1 - D(G(z))) \right\} = \underset{G}{min} \left\{ \log D(x) + \log(1 - D(G(z))) \right\}. \tag{7}$$

In order to obtain a single objective function for the GAN, we combine Eqns. (6) and (7) and take the expectation over the whole dataset. The resulting objective function for the GAN, which is inspired by the cross-entropy loss, is given by:

$$\min_G \max_D \left\{ \mathbb{E}_{x \sim P_{data}(x)}[\log D(x)] + \mathbb{E}_{z \sim P_z(z)}[\log(1 - D(G(z)))] \right\}. \tag{8}$$

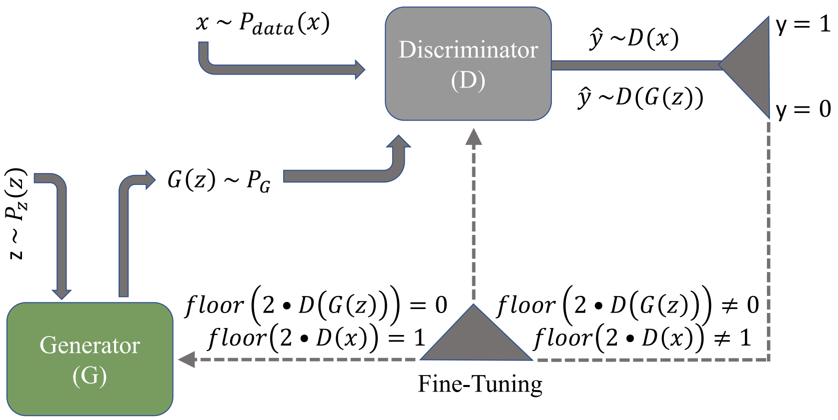

Fig. S1 Schematic of a generative adversarial network (GAN). The Generator (G) and Discriminator (D) are two neural networks that compete with each other during the training process, with the end result being that G can produce fake samples from a distribution that matches the distribution of the real samples. $D(x)$ and $D(G(z))$ provide the reconstructed label $\hat{y}$ (i.e. the output of the Discriminator network), which represents the probability of the sample being real rather than fake. If $\hat{y}$ is greater than (less than) 0.5, the Discriminator classifies the sample as real (fake). If D is correct (incorrect), then the weights of G (D) are fine-tuned through backpropagation.

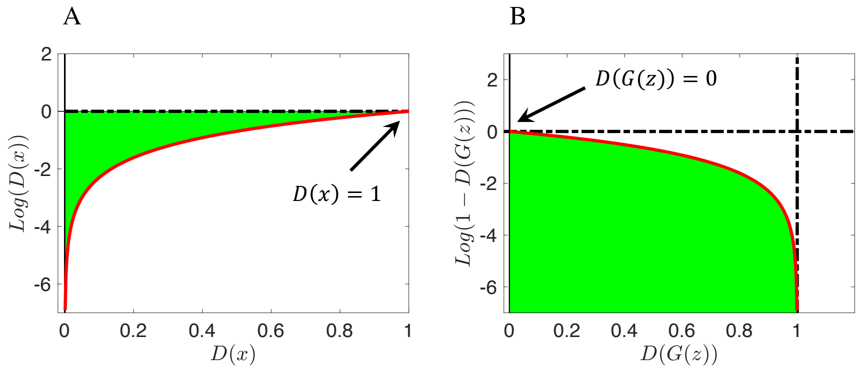

Fig. S2 Visualization of the components of the GAN objective function (Eqn. (8)). (A) Discriminator loss function (Eqn. (4)). $D(x)$ is the probability that x came from the real data rather than P_g. **(B)** Generator loss function given (Eqn. (5)). $D(G(z)))$ is the probability that $G(z)$ came from P_g rather than P_{data}. The green shading identifies the possible regions for these two probabilities.

3 Supplementary Results

3.1 Synthetic target methodology

As we are dealing with 5 parameters in our mechanistic model, we design different target data scenarios with 5 choose k parameters distinguishing two different groups of target data.

5 choose 0: In this scenario, since $k = 0$, we only have one target group. All 5 parameters are drawn from a normal distribution with a mean μ and standard deviation $\mu/8$, where μ is the value of that parameter in the optimized DE model parameter set (DE-MG-Vmnat). There is only one case to consider in this scenario.

5 choose 1: In this scenario, since $k = 1$, we choose one of the 5 parameters to draw from a different distribution for the Group 1 (G1) and Group 2 (G2) target datasets. For that parameter, we draw the G1 samples from a normal distribution with a mean of 0.5μ and the G2 samples from a normal distribution with a mean of 1.5μ. For both groups the normal distribution has a standard deviation of $\mu/8$, where again μ is the value of that parameter in the optimized DE model. There are 5 different cases in this scenario, since there are 5 parameters that can be chosen to distinguish G1 and G2.

5 choose k***:*** Here we consider the scenarios $k \in \{2, 3, 4, 5\}$. The number of cases for each value of k can be computed from Eqn. (9). The 2^{k-1} term reflects the number of different ways k parameters can be different between G1 and G2. For example, suppose $k = 3$, and the 3 parameters chosen to be distributed differently between G1 and G2 are *g-Na-T*, *g-Ca-H*, and *g-K-DR*. There are 2^2 different possibilities: (1) all 3 parameters are low in G1 and high in G2 – denoted L-H, L-H, L-H; (2) *g-Na-T* is high in G1 and *g-Ca-H*, *g-K-DR* are low in G1 – denoted H-L, L-H, L-H; (3) *g-Na-T* low in G1, *g-Ca-H* high in G1, and *g-K-DR* low in G1 – denoted L-H, H-L, L-H; and (4) *g-Na-T*, *g-Ca-H* low in G1 and *g-K-DR* high in G1 – denoted L-H, L-H, H-L. We do not have to simulate possibilities such as H-L, H-L, H-L or L-H, H-L, H-L, since they are equivalent to possibilities (1) and (2) listed above, respectively, just with the

labels swapped for G1 and G2 swapped.

$$\binom{n}{k} \times 2^{k-1}, \quad k = 1, \cdots, 5 \tag{9}$$

3.2 Kolmogorov Smirnov tests

We performed Kolmogorov Smirnov tests (KS-tests) to compare the cGAN samples to the ground truth target samples for all the different 5 choose k synthetic target scenarios. The null hypothesis for these tests is that the two samples are from the same probability distribution. The plots in Figs. S1-S6 represent the results of these tests, where black indicates the null hypothesis is rejected (p-value $\leq$ 0.01) and peach indicates the null hypothesis is not rejected (p-value $>$ 0.01).

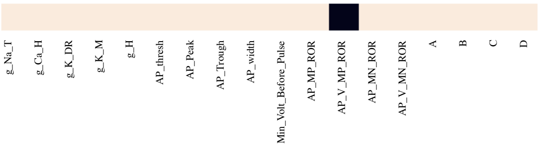

Fig. S1 5 choose 0 - KS tests for cGAN samples versus target data samples. Peach color indicates failure to reject the null hypothesis that the cGAN samples and synthetic target data samples are from the same distribution.

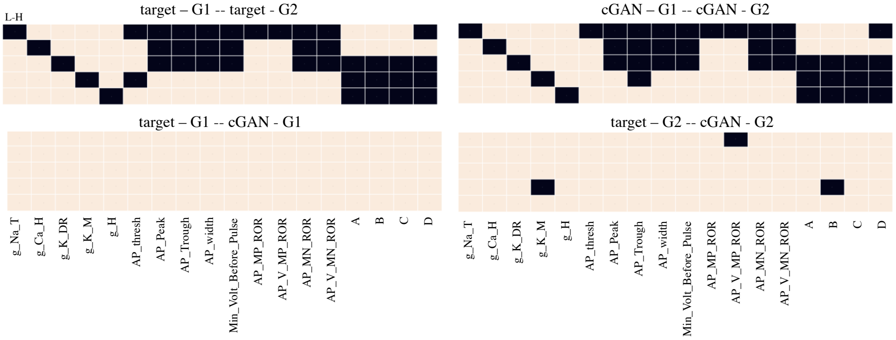

Fig. S2 5 choose 1 - KS tests. ***Top left*** - KS tests on two groups of targets (i.e. G1 (G2) with low - **L** (high - **H**) values of the parameter). ***Top right*** - KS test on cGAN samples corresponding to the two groups of target data (i.e. cGAN-G1 (cGAN-G2) with low - **L** (high - **H**) values of the parameter). ***Bottom left*** - KS test of cGAN-G1 versus target-G1. ***Bottom right*** - KS test of cGAN-G2 versus target-G2.

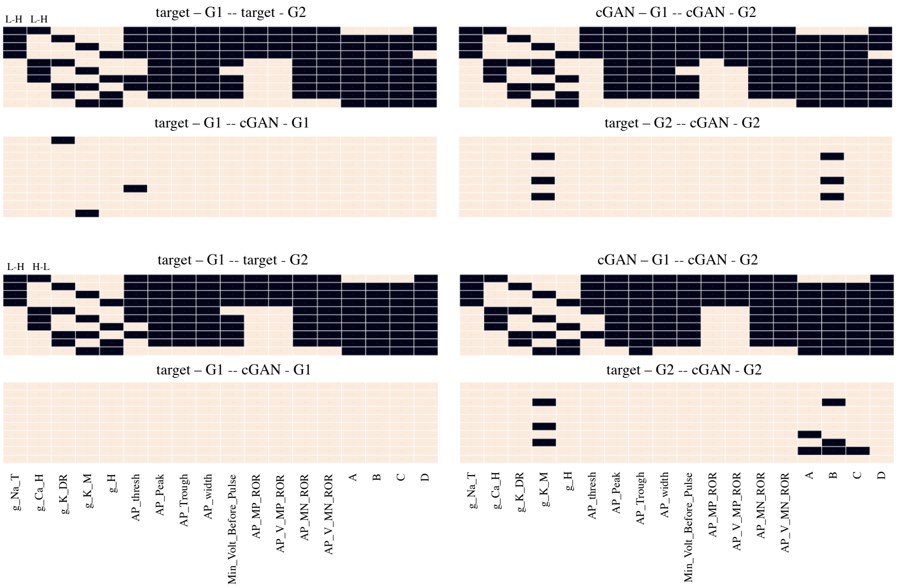

Fig. S3 5 choose 2 - KS tests. Panels are arranged in a similar fasion as Fig. S2. Top: L-H, L-H. Bottom: L-H, H-L.

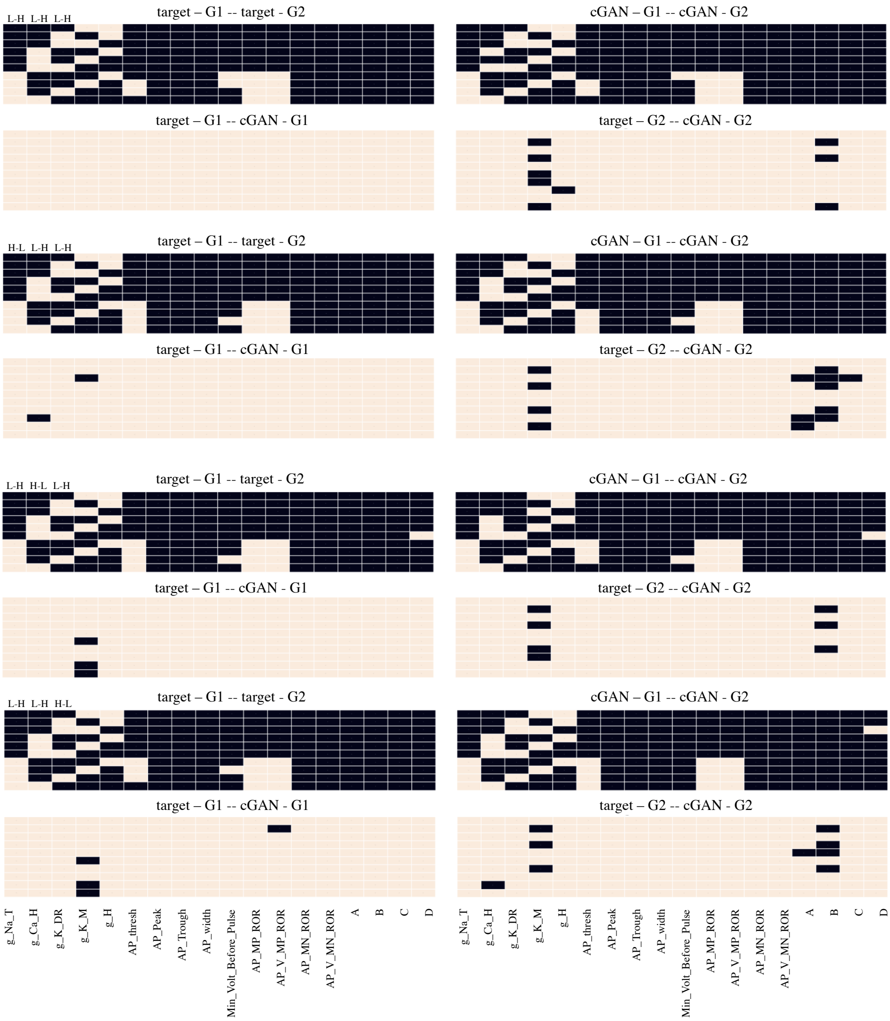

Fig. S4 5 choose 3 - KS tests. Panels are arranged in a similar fashion as Fig. S2. From top to bottom: ***(1) L-H L-H L-H***, ***(2) H-L L-H L-H***, ***(3) L-H H-L L-H***, ***(4) L-H L-H H-L***.

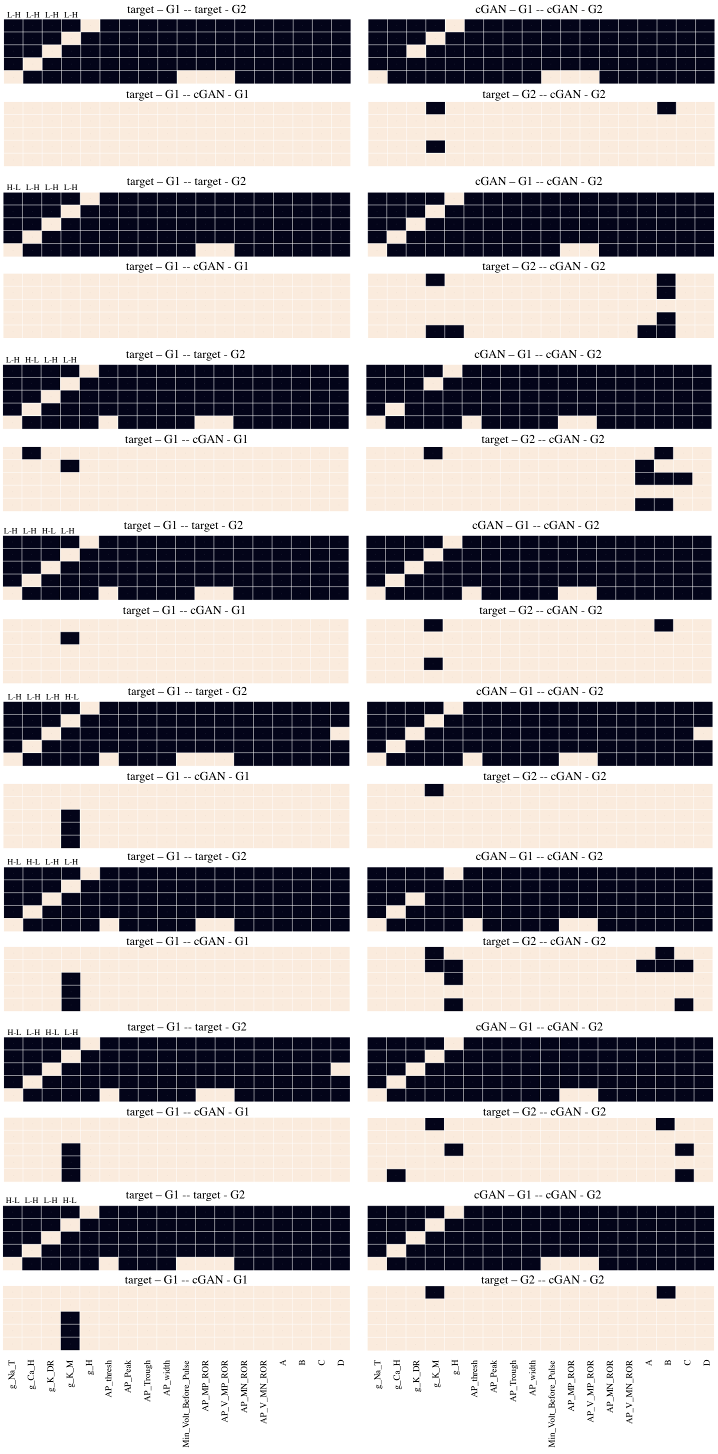

Fig. S5 5 choose 4 - KS tests. Panels are arranged in a similar fashion as Fig. S2. From top to bottom: *(1)* ***L-H L-H L-H L-H***, *(2)* ***H-L L-H L-H L-H***, *(3)* ***L-H H-L L-H L-H***, *(4)* ***L-H L-H H-L L-H***, *(5)* ***L-H L-H L-H H-L***, *(6)* ***H-L H-L L-H L-H***, *(7)* ***H-L L-H H-L L-H***, *(8)* ***H-L L-H L-H H-L***.

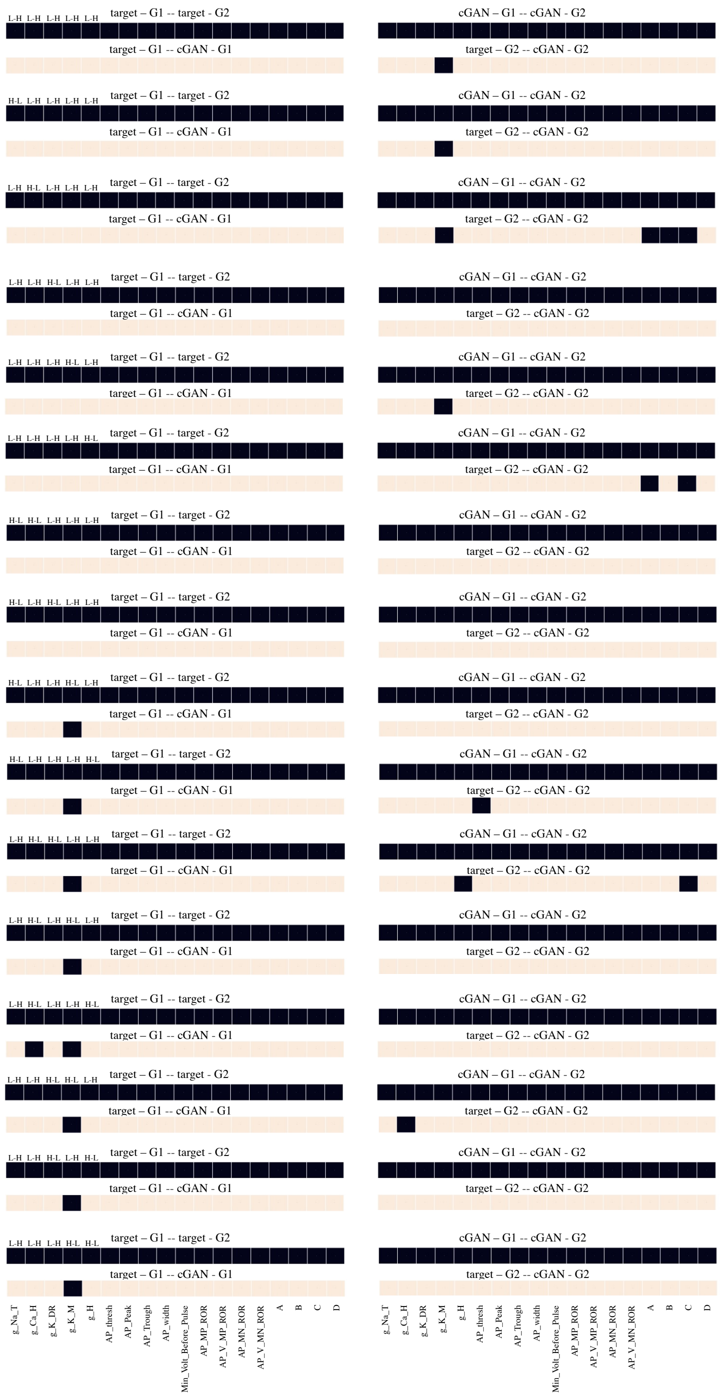

Fig. S6 5 choose 5 - KS tests. - Panels are arranged in a similar fashion as Fig. S2. From top to bottom: *(1) L-H L-H L-H L-H L-H*, *(2) H-L L-H L-H L-H L-H*, *(3) L-H H-L L-H L-H L-H*, *(4) L-H L-H H-L L-H L-H*, *(5) L-H L-H L-H H-L L-H*, *(6) L-H L-H L-H H-L L-H H-L*, *7) H-L H-L L-H L-H L-H*, *(8) H-L L-H H-L L-H L-H*, *(9) H-L L-H L-H H-L L-H*, *(10) H-L L-H L-H L-H H-L*, *(11) L-H H-L H-L L-H L-H*, *(12) L-H H-L L-H H-L L-H*, *(13) L-H H-L L-H L-H H-L*, *(14) L-H L-H H-L H-L L-H*, *(15) L-H L-H H-L L-H H-L*, *(16) L-H L-H L-H H-L H-L*.

5-choose-1

True	2/90
0/90	3/90

Total = 5/270

5-choose-2

True	7/360
3/360	15/360

Total = 25/1080

5-choose-3

True	9/720
9/720	38/720

Total = 56/2160

5-choose-4

True	7/720
15/720	41/720

Total = 63/2160

5-choose-5

True	0/288
9/288	13/288

Total = 22/864

Fig. S7 Summary of KS test results for all 5 choose k synthetic target data cases. The demoninators are the total number of tests. **Top left quadrants:** These KS tests are taken to be the ground truth as they compared target G1 versus target G2. **Top right quadrants:** These KS tests compared cGAN-G1 versus cGAN-G2. The numerator is the number of tests for which cGAN-G1 versus cGAN-G2 returned a different result than target G1 versus target G2. **Bottom left quadrants:** These KS tests compared target-G1 versus cGAN-G1. The numerator are the number of those tests for which the null hypothesis was rejected. **Bottom left quadrants:** These KS tests compared target-G2 versus cGAN-G2. The numerator are the number of those tests for which the null hypothesis was rejected.

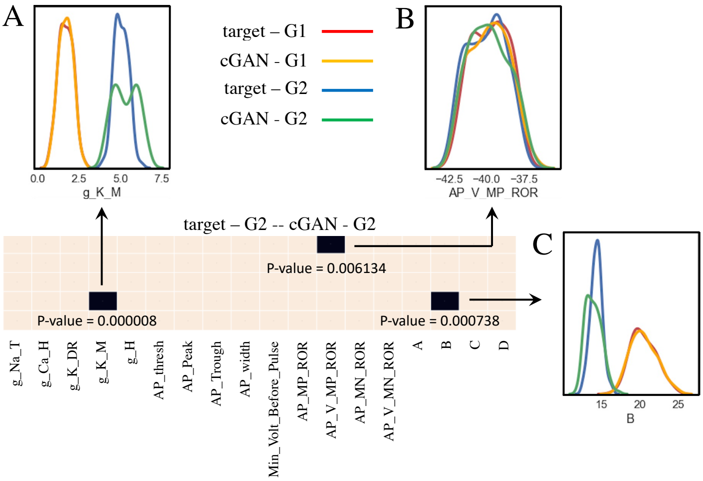

Fig. S8 5 choose 1 - mis-rejected KS tests of cGAN-G2 versus target-G2. (A) The KS test rejected the null-hypothesis (p-value = 0.000008 < 0.01) between the target-G2 and cGAN-G2 distribution for the g-K-M parameter. **(B)** The KS test rejected the null-hypothesis (p-value = 0.006134 < 0.01) between the target-G2 and cGAN-G2 distribution for the AP-V-MP-ROR feature. **(C)** The KS test rejected the null-hypothesis (p-value = 0.000738 < 0.01) between the target-G2 and cGAN-G2 distribution for the HP-B feature.

3.3 Output of cGAN samples in feature space for synthetic target data

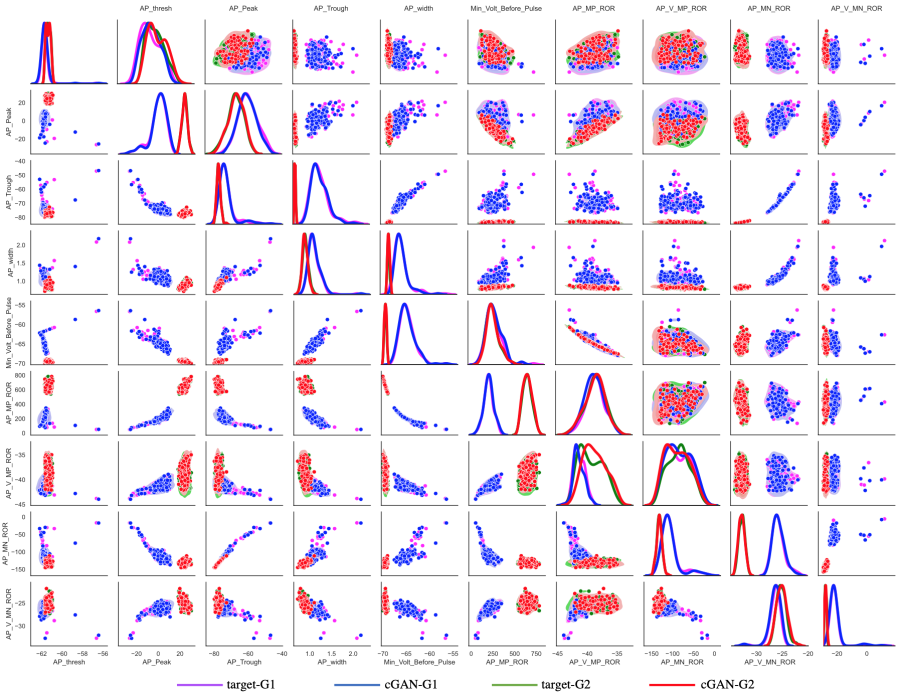

Fig. S9 Performance of cGAN on synthetic targets from 2 groups with distinct parameter structures - AP features. KDE plots (main diagonals) and scatter plots (lower and upper triangles) for Group 1 (G1) target data (magenta), Group 2 (G2) target data (green), cGAN samples for G1 (blue) and cGAN samples for G2 (red). *Lower main diagonal and lower triangle* - only 1 parameter (g_{NaT}) is distributed differently in the G1 target data than in the G2 target data, and the other 4 parameters have the same distribution in the G1 and G2 target data. We refer to this scenario as "5 choose 1" in the Supplementary Methods. *Upper main diagonal and upper triangle* - Four parameters (all parameters except g_{NaT}) are distributed differently in the G1 target data than in the G2 target data. We refer to this scenario as "5 choose 4" in the Supplementary Methods.

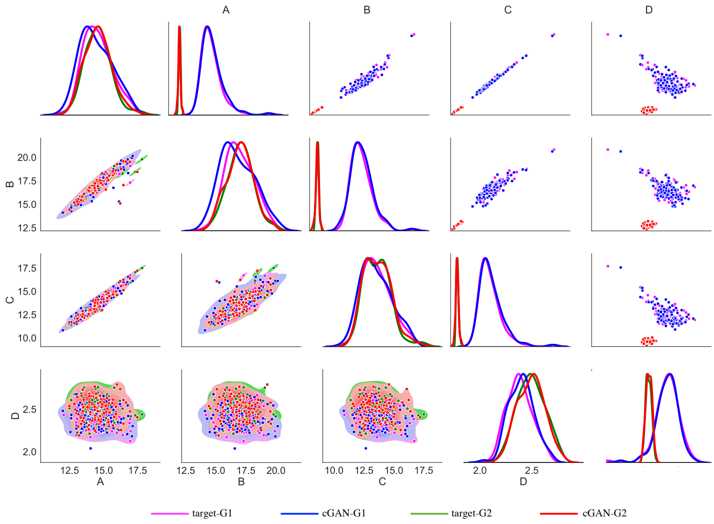

Fig. S10 Performance of cGAN on synthetic targets from 2 groups with distinct parameter structures - HP features. KDE plots (main diagonals) and scatter plots (lower and upper triangles) for Group 1 (G1) target data (magenta), Group 2 (G2) target data (green), cGAN samples for G1 (blue) and cGAN samples for G2 (red). *Lower main diagonal and lower triangle* - only 1 parameter (g_{NaT}) is distributed differently in the G1 target data than in the G2 target data, and the other 4 parameters have the same distribution in the G1 and G2 target data. *Upper main diagonal and upper triangle* - Four parameters (all parameters except g_{NaT}) are distributed differently in the G1 target data than in the G2 target data.

3.4 Output of cGAN samples in feature space for experimental target data

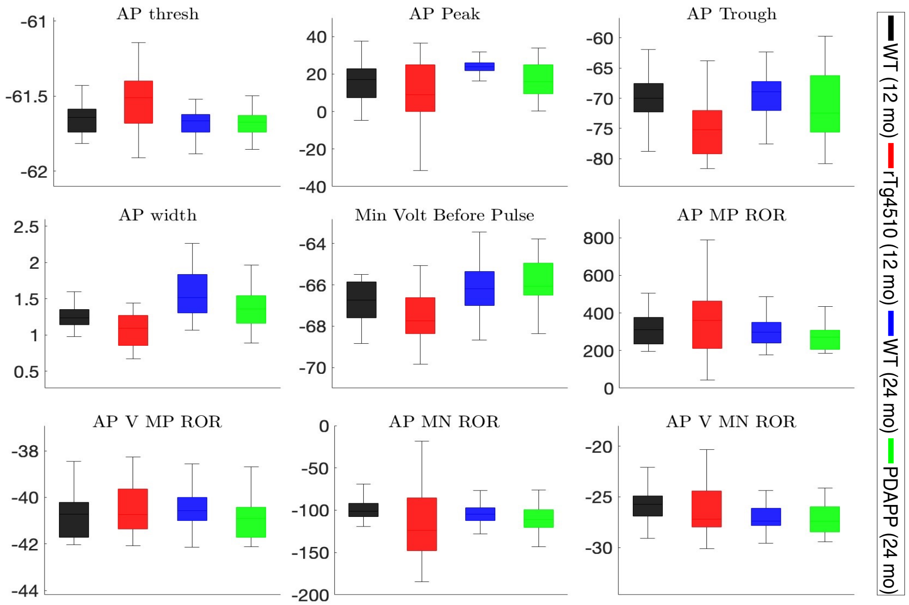

Fig. S11 Box and whisker plots of the action potential (AP) features extracted from the mechanistic model voltage traces obtained by pushing forward the cGAN parameter samples with experimental target data.

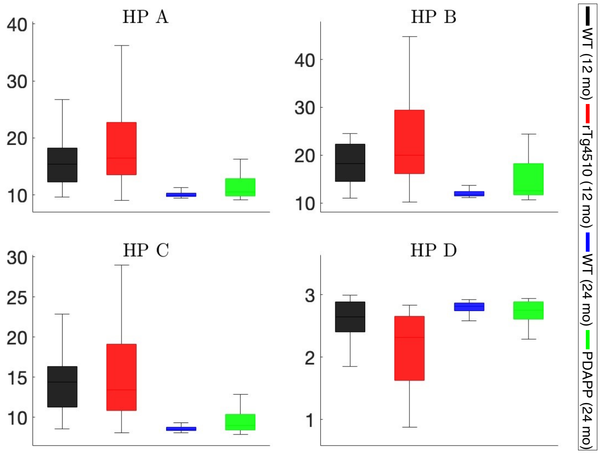

Fig. S12 Box and whisker plots of the membrane hyperpolarization (HP) features extracted from the mechanistic model voltage traces obtained by pushing forward the cGAN parameter samples with experimental target data.

References

[1] Nowacki, J., Osinga, H.M., Brown, J.T., Randall, A.D., Tsaneva-Atanasova, K.: A unified model of CA1/3 pyramidal cells: an investigation into excitability. Progress in Biophysics and Molecular Biology **105**(1-2), 34–48 (2011)

[2] Booth, C.A., Witton, J., Nowacki, J., Tsaneva-Atanasova, K., Jones, M.W., Randall, A.D., Brown, J.T.: Altered intrinsic pyramidal neuron properties and pathway-specific synaptic dysfunction underlie aberrant hippocampal network function in a mouse model of tauopathy. Journal of Neuroscience **36**(2), 350–363 (2016)

[3] Price, K., Storn, R.M., Lampinen, J.A.: Differential Evolution: a Practical Approach to Global Optimization. Springer, 53851, Lappeenranta (2006)

[4] Storn, R., Price, K.: Differential evolution–a simple and efficient heuristic for global optimization over continuous spaces. Journal of Global Optimization **11**(4), 341–359 (1997)

[5] Sobol, I.M.: Global sensitivity indices for nonlinear mathematical models and their monte carlo estimates. Mathematics and Computers in Simulation **55**(1-3), 271–280 (2001)

[6] Tosin, M., Côrtes, A., Cunha, A.: A Tutorial on Sobol' Global Sensitivity Analysis Applied to Biological Models. Networks in Systems Biology, 93–118 (2020)

[7] Konakli, K., Sudret, B.: Global sensitivity analysis using low-rank tensor approximations. Reliability Engineering & System Safety **156**, 64–83 (2016)

[8] Iwanaga, T., Usher, W., Herman, J.: Toward SALib 2.0: Advancing the accessibility and interpretability of global sensitivity analyses. Socio-Environmental Systems Modelling **4**, 18155–18155 (2022)

[9] Herman, J., Usher, W.: SALib: An open-source Python library for sensitivity analysis. Journal of Open Source Software **2**(9), 97 (2017)

[10] Jaxa-Rozen, M., Kwakkel, J.: Tree-based ensemble methods for sensitivity analysis of environmental models: A performance comparison with sobol and morris techniques. Environmental Modelling & Software **107**, 245–266 (2018)

[11] Lence, B.J., Takyi, A.: Data requirements for seasonal discharge programs: An application of a regionalized sensitivity analysis. Water Resources Research **28**(7), 1781–1789 (1992)

[12] Hoff, P.D.: A First Course in Bayesian Statistical Methods vol. 580. Springer, NY10013, USA (2009)

[13] Gelman, A., Carlin, J.B., Stern, H.S., Rubin, D.B.: Bayesian Data Analysis. Chapman and Hall/CRC, 6000 Broken Sound Parkway NW, Suite 300 (1995)

[14] Brooks, S., Gelman, A., Jones, G., Meng, X.-L.: Handbook of Markov Chain Monte Carlo. CRC Press, 6000 Broken Sound Parkway NW, Suite 300 (2011)

[15] Brooks, S.: Markov chain Monte Carlo method and its application. Journal of the Royal Statistical Society: Series D (The Statistician) **47**(1), 69–100 (1998)

[16] Cohen, J.: The effect size. Statistical power analysis for the behavioral sciences, 77–83 (1988)

[17] Sawilowsky, S.S.: New effect size rules of thumb. Journal of modern applied statistical methods **8**(2), 26 (2009)
